# Communication sheet eases barriers for Japanese patients and health professionals

**DOI:** 10.1186/s12913-022-08371-x

**Published:** 2022-07-30

**Authors:** Kento Sonoda, Teiichi Takedai, Cynthia Salter

**Affiliations:** 1 Department of Family Medicine, University of Pittsburgh Medical Center Shadyside, 5215 Centre Avenue, Pittsburgh, PA 15232 USA; 2 Department of Family and Community Medicine, Saint Louis University, Saint Louis, MO USA; 3 Graduate School of Public Health, University of Pittsburgh, Pittsburgh, PA USA

**Keywords:** Healthcare disparities, Cultural competency, Delivery of health care, Health services accessibility, Healthcare delivery, Communication

## Abstract

**Background:**

Language and cultural barriers can affect healthcare outcomes of minority populations. However, limited data are available on communication tools developed to address health disparities resulting from language and cultural barriers. Our study aimed to reduce communication barriers between Japanese patients and non-Japanese-speaking clinic staff by developing a Japanese-English Communication Sheet (JECS) to create more equitable clinical environments for Japanese patients in ambulatory care.

**Methods:**

This study was conducted at a family health center in a United States urban setting, in the city of Pittsburgh, between November 2019 and August 2020. This study included Japanese adult patients who had health care office visits with one of two Japanese-speaking physicians and who completed a survey about the JECS. The JECS, written in Japanese and English, targets common sources of confusion by presenting common health questions, written in Japanese, and by explaining differences between common healthcare processes in Japan and the United States. Clinic staff who used the JECS with Japanese-speaking patients also were surveyed about the tool.

**Results:**

Sixty Japanese patients met inclusion criteria and completed the survey. More than half of participants found the JECS useful, and those with self-reported limited English proficiency were most likely to report that the JECS was useful (*p* = 0.02). All nine non-Japanese speaking staff surveyed found the sheet helpful.

**Conclusions:**

The JECS is a useful communication tool for addressing common barriers faced by Japanese patients seeking care at an American health center where Japanese-speaking physicians work but no clinic staff speak Japanese. A focused communication sheet can facilitate communication between patients and clinic staff and also reduce health inequities resulting from linguistic and cultural barriers. Additionally, using a communication sheet can advance quality and safety of patient care at the individual and institutional level.

**Supplementary Information:**

The online version contains supplementary material available at 10.1186/s12913-022-08371-x.

## Background

The most recent US census revealed that more than 60 million people in the United States speak a language other than English at home [1]. Additionally, the World Migration Report 2020 estimated the number of international immigrants at 272 million [2]. In the United States, language and cultural barriers can negatively affect non-English-speaking patients regarding timely access to healthcare, safety, and health outcomes [3–7]. Professional interpreter services can help health care professionals reduce the disparity gap [8–11], but such services may not always be available. A survey study in Canada, for example, found that most family physicians do not take advantage of professional interpreters although communication difficulties are a key barrier to the management of immigrant patients [12]. An earlier qualitative study found that general practitioners face considerable professional uncertainty and occupational stress when managing diverse populations, often due to cultural differences [13]. A 2020 scoping review revealed that when language barriers between patients and nurses existed, nurses had difficulty accessing interpreters, experienced increased workload, and lacked cultural competency skills [14].

In the United States, patient care at a clinic begins with “rooming,” where clinic staff show patients to examination rooms and interact with patients to obtain brief medical information before patients see their physicians. Since two Japanese-speaking physicians work at our clinic in Pittsburgh, (United States), many Japanese-speaking patients commonly seek primary care there. In a 2021 Education First English Proficiency Index Report, Japan was ranked low, at 78, out of 112 countries [15]. Japanese patients do not need professional interpreter services while seeking care from Japanese-speaking physicians, however, the initial rooming process with non-Japanese-speaking staff requires communication in English. One of the study authors, a faculty physician originally from Japan who has worked at the health center for 12 years, noted significant delays in the rooming process for Japanese-speaking patients by clinic staff who do not speak Japanese, even when telephone translation service may have been used. Through his work with the Japanese community in Pittsburgh, this physician knew that most Japanese patients have completed high school or higher-level education, have middle or high socioeconomic status, and can read Japanese. Many come to Pittsburgh for higher education or professional opportunities and demonstrate high English proficiency, often due to requirements of their degree program or employer. However, their partners and other family members tend to have lower English proficiency, as is common in the general population in Japan. The first author of this study was a resident physician who noted the same problems of delayed and stunted communication between Japanese-speaking patients and non-Japanese-speaking clinic staff, which often delayed start times for patient appointments. These common communication issues prompted the creation of a communication tool to address this unique gap.

A few previous studies have explored the development and use of communication tools for patients with limited English proficiency to address their language barriers [16, 17]. For example, in the U.S., which has a large population that speaks only Spanish, a group in Texas developed an audio-recorded Question Prompt List in Spanish for use by non-Spanish-speaking clinicians with Latino parents whose infants were in the Neonatal Intensive Care Unit [16]﻿. In Germany, researchers developed a digital communication assistance tool (DCAT) for 19 different languages and dialects [17], and another German project developed a language app for assisting paramedics to care for non-German-speaking patients in acute situations [18].

However, none of the communication tools addressed cultural differences or unfamiliarity with the healthcare system, and few data are available on strategies in the United States to manage language and cultural barriers between clinic staff and patients with limited English proficiency and lack of familiarity with the healthcare system. This study aimed to reduce communication barriers between Japanese patients and non-Japanese-speaking health professionals by implementing a new, easily accessible communication tool, called the Japanese-English Communication Sheet (JECS), created for this study.

## Methods

### Participants and setting

This single-institutional observational study was conducted among Japanese patients who seek health care from two Japanese-speaking physicians but who also interacted with other clinic staff who do not speak Japanese. The project was implemented and assessed for ten months between November 2019 and August 2020 at a family health center in an urban Pittsburgh setting in the United States. We included Japanese patients aged 20 years or older, and parents who accompanied their children for any types of visits with two Japanese-speaking physicians. Patients who were not seen by Japanese-speaking physicians and patients who received a telemedicine visit were excluded. Clinic staff who interacted with patients during rooming also completed an anonymous post-implementation questionnaire in August 2020 to assess their awareness and use of the JECS and its helpfulness for patient communication. Front desk staff were excluded from this study. The institutional Quality Improvement Review Committee approved this study.

### Implementation

The first two authors developed a Japanese-English Communication Sheet (JECS), drawing upon the second author’s 12-year experience with the Japanese-speaking patient population and after completing interviews with all clinic staff. The JECS was fine-tuned through an iterative process with multiple native speakers in Japanese and English, under the supervision of the institutional senior director of consumer engagement. We developed the JECS, written in Japanese and English, by modifying routine rooming questions from clinic staff to patients before physician encounters, based on common sources of confusion during rooming process. The JECS explains differences between care processes in Japan and the United States, including differences in culture, the use of electronic prescription process for pharmaceuticals (common in the U.S. but not in Japan), and procedures for obtaining screening tests. The JECS covers a variety of basic medical information such as date of birth, reason for office visit, pain level, allergic histories, and PHQ-2/GAD-2 screening tests. Under a preferred pharmacy section, the JECS includes the following explanation, written in Japanese, “Electronic prescription is available in the United States. Please tell us a name of pharmacy close to you”. This statement helps Japanese patients better understand the reason why healthcare staff ask about their preferred pharmacy.

The JECS was designed to be used by non-Japanese-speaking clinic staff during rooming for office visits with Japanese patients. The overall goal was to reduce communication barriers that added to rooming time by assisting the non-Japanese-speaking staff to easily collect key information necessary for the rooming process. However, the JECS was also developed for bridging the knowledge gap resulting from cultural differences such as an e-scribe prescription system, which is not available in Japan, or U.S. style description of date of birth, which differs from traditional Japanese.

The JECS was piloted for one month; follow-up interviews were conducted with all clinic staff, resulting in the addition of a question about last menstrual period and instructions for completing urine tests, based on staff feedback.

### Data collection and analysis

Data were collected via two surveys: one completed by patients and another completed by non-Japanese-speaking clinic staff. At the end of each office visit, participating patients completed questionnaires that asked about the usefulness of the JECS and their willingness to use the JECS at their next health center visit, with possible responses of Yes, No, and Unsure. Participants also self-evaluated their English language proficiency using the following four levels: “Can’t speak at all”, “Can speak a little”, “Can speak fairly well”, and “Can speak fluently”. Clinic staff also evaluated the helpfulness of the JECS, using Likert scale responses 1 to 5 (1 = slightly helpful, 5 = very helpful) (See Additional File 1).

Data analysis included calculating descriptive statistics for the percentage of respondents who found the JECS useful as well as performing a Chi-Square test of association to explore associations between English proficiency level and perceived usefulness of the JECS. For Chi Square analysis, we created two categories by combining participants’ self-assessment of their English skills: Advanced level, which included self-assessment of “Can speak fairly well” and “Can speak fluently”; and Basic level, which included self-assessment of “Can’t speak at all” and “Can speak a little.” We made “Usefulness of the JECS” into a binary variable by combining “unsure” and “no” into one category of “Not Useful or Unsure”. We completed the Chi Square analysis comparing the two categorical variables, English Language Skill Level and Usefulness of the JECS (significance level 0.05, 1 degree of freedom).

## Results

### Patients

Sixty Japanese patients attended primary care appointments with the two Japanese-speaking physicians during the study period, and nine clinic staff used the JECS to communicate with them. Out of 60 patient respondents, 51 were female (85%), 43 were age 30–39 years old (71.7%), and 31 attended the health center for Well Child Care (51%) (Table 1). More than half of participants found the JECS useful and reported willingness to use the JECS again at the next visit (Table 2). Around 40% (26/60) of the participants reported limited English proficiency (Table 3). Chi Square testing found that participants with basic English level (“Can speak a little” or “Can’t speak at all”) were significantly more likely to find the JECS useful, compared with those with advanced English level (“Can speak fairly well” or “Can speak fluently”) (*p* = 0.02) (Table 4).

**Table 1:** Characteristics of participants and types of office visits (*n* = 60)

Variable	Number (n)	Proportion (%)
**Gender**		
Women	51	85
Men	9	15
**Age group (Years)**		
20 ~ 29	0	0
30 ~ 39	43	71.7
40 ~ 49	11	18.3
50 ~ 59	2	3.3
60 ~ 69	1	1.7
70 ~ 79	1	1.7
80 ~ 89	2	3.3
**Visit type**		
Well Child Care	31	51.7
Sick Visit	16	26.7
Annual Exam	7	11.7
Obstetric Care	6	10

Annual Exam includes annual physical exam and annual gynecological exam

**Table 2:** Usefulness based on english proficiency level (*n* = 60)

Variables	Number (n)	Proportion (%)
**Usefulness**		
Yes	34	56.7
No	11	18.3
Unsure	15	25
**Willingness to use again**		
Yes	35	58.3
No	13	21.7
Unsure	12	20

*P* = 0.02%

**Table 3:** Self-reported english proficiency level (*n* = 60)

	**Self-reported English proficiency level**	**Number (n)**	**Proportion (%)**
Advanced level	Can speak fluently	21	35
	Can speak fairly well	13	21.7
Basic level	Can speak a little	24	40
	Can't speak at all	2	3.3

**Table 4:** Usefulness based on self-reported english proficiency level (*n* = 60)

	**Basic English level**	**Advanced English level**	**Number (n)**	**Proportion (%)**
Useful	19	15	34	56.7
Not Useful or Unsure	7	19	26	43.3
	26	34	60	

### Clinic staff

Nine clinic staff answered a post-implementation survey (100% response rate), including six registered nurses, two medical assistants, and one registered radiologic technologist. All clinic staff used the JECS and found the JECS a helpful tool (Table 5).

**Table 5:** Helpfulness of the JECS from clinic staff (*n* = 10)

Variables	Number (n)	Proportion (%)
**Helpfulness**		
Yes	9	100
No	0	0
Unsure	0	0
**Likert scale of helpfulness (Scale 1–5)**		
1	0	0
2	0	0
3	0	0
4	2	22.2
5	7	77.8

Likert scale for helpfulness: 1 = slightly helpful; 5 = very helpful

## Discussion

This study found that the JECS, a straightforward and relatively simple communication tool made up of a laminated sheet of paper, printed in Japanese and English with specific comments to explain cultural gaps and differences in health care between the U.S. and Japan, helped Japanese patients and non-Japanese-speaking clinic staff communicate with each other. The idea of the JECS can be applied to other languages and cultures to help patients who have moved to a new country where they are not familiar with the language, culture, or healthcare system although it is only applicable to those with literacy skills in their native language. Health care professionals could even create several versions, adjusted for different types of office visit to meet unique patient needs. This cost-effective method also can help ensure the accuracy of patient information, including date of birth, chief complaints, office visit types, and screening for depression and anxiety. For example, we learned from conversations with Japanese patients seeking mental health care that Japanese patients might conceptualize the screening questions for depression and anxiety better in their native language, even when they completely understand the meaning of the screening questions in English.

To reduce health disparities for minority populations, strategies for improving communication as well as cross-cultural communication skills among health care professionals should be more fully explored. From the clinicians’ perspective, developing this type of communication sheet can help empower clinicians in all three elements of cultural competency, including knowledge, attitudes, and skills. The JECS, for example, helped health care professionals better understand patient context (knowledge), respect cultural differences (attitudes), and achieve better cross-cultural communication (skills). To improve cross-cultural communication, this communication sheet can play a supplemental role of being used as a training material in variety of activities such as lectures, workshop, mentoring, and supervision. This type of communication sheet can contribute to the ongoing learning process of cross-cultural communication, at both the individual and organizational level.

This study includes several limitations. First, Japanese patients might have rated the JECS more highly since they were aware that two Japanese-speaking physicians would review their ratings later (social acceptability bias). Additionally, the JECS was not used for telemedicine visits, and due to the COVID-19 infection pandemic, many patient visits after April 2020 were via telemedicine. Also, the survey results of this assessment were not triangulated with in-depth interviews with patients or clinic staff because of resource constraints. We conducted only brief interviews with clinic staff at the inception of this project, for assessment of a pilot use, and at the end of post-implementation period instead of in-depth interviews. Lastly, English proficiency level was measured by self-evaluation, which may not accurately represent participants’ actual language proficiency level.

## Conclusions

This study found that the JECS was useful for most Japanese patients, especially those with limited English proficiency. Participants with self-reported limited English proficiency were significantly more likely to find the JECS useful than participants with higher levels of English proficiency. Developing a communication sheet to address linguistic and cultural barriers can help patients and clinic staff communicate with each other, which can create more equitable clinical environments for providing care to minority populations. Improved communication can advance the quality and safety of patient care at the individual and institutional level. Additionally, clinicians can improve their cross-cultural communication skills by assessing their own knowledge, attitudes, and skills in the process of developing a communication sheet. Further research on how to address barriers to health care related to languages and cultures should be explored.

## Supplementary Information

**Additional file 1.**

## Data Availability

The datasets used and/or analysed during the current study are available from the corresponding author on reasonable request.

## Abbreviations

ACGME: The Accreditation Council for Graduate Medical Education
JECS: Japanese-English Communication Sheet
