# Supplementary material for: Communication sheet eases barriers for Japanese patients and health professionals
Source: BMC Health Serv Res. 2022 Jul 30;22:976. doi: 10.1186/s12913-022-08371-x (PMC9339196; doi:10.1186/s12913-022-08371-x)
Supplement: Supplementary file 1 — Additional file 1. [file 12913_2022_8371_MOESM1_ESM.docx]

“Communication Sheet for Japanese Patients” QI Project

Kento Sonoda, Teiichi Takedai

Questionnaires for health professionals (post**-**implementation)

How long have you worked at FHC Shadyside? ____ years (If within 1 year 🡪 _____ months)

Please circle your medical credential(s). RN / LPN / MA / Other(s) __

Are you aware that there is a “Communication Sheet” to help communications between Japanese patients and nurses?

- YES / NO / Not Sure

If you are aware of the “Communication Sheet”, have you ever used the “Communication Sheet” to communicate with Japanese patients?

- YES / NO / Not Sure

Do you think the “Communication Sheet” for Japanese patient is a helpful tool?

- YES / NO / Not Sure

If yes, please rate the helpfulness of “Communication Sheet” on the scale of 1-5, 1 being slightly helpful and 5 being very helpful.

- 1 2 3 4 5

Comments;
